# Mortality from Cardiovascular Diseases in the Elderly: Comparative Analysis of Two Five-year Periods

**DOI:** 10.5935/abc.20150096

**Published:** 2015-10

**Authors:** Grasiela Piuvezam, Wilton Rodrigues Medeiros, Andressa Vellasco Costa, Felipe Fonseca Emerenciano, Renata Cristina Santos, Danilo Silveira Seabra

**Affiliations:** Universidade Federal do Rio Grande do Norte, Natal, RN – Brazil

**Keywords:** Cardiovascular Diseases / mortality, Cardiovascular Diseases / epidemiology, Comparative Study, Aged

## Abstract

**Background:**

Cardiovascular diseases are the leading cause of death in Brazil. The better understanding of the spatial and temporal distribution of mortality from cardiovascular diseases in the Brazilian elderly population is essential to support more appropriate health actions for each region of the country.

**Objective:**

To describe and to compare geospatially the rates of mortality from cardiovascular disease in elderly individuals living in Brazil by gender in two 5-year periods: 1996 to 2000 and 2006 to 2010.

**Methods:**

This is an ecological study, for which rates of mortality were obtained from DATASUS and the population rates from the Brazilian Institute of Geography and Statistics (Instituto Brasileiro de Geografia e Estatística). An average mortality rate for cardiovascular disease in elderly by gender was calculated for each period. The spatial autocorrelation was evaluated by TerraView 4.2.0 through global Moran index and the formation of clusters by the index of local Moran-LISA.

**Results:**

There was an increase, in the second 5-year period, in the mortality rates in the Northeast and North regions, parallel to a decrease in the South, South-East and Midwest regions. Moreover, there was the formation of clusters with high mortality rates in the second period in Roraima among females, and in Ceará, Pernambuco and Roraima among males.

**Conclusion:**

The increase in mortality rates in the North and Northeast regions is probably related to the changing profile of mortality and improvement in the quality of information, a result of the increase in surveillance and health care measures in these regions.

## Introduction

Noncommunicable Diseases (NCD), according to the worldwide trend of recent decades, currently determine the majority of causes of death in Brazil, changing the profile of diseases that occur in the population, being higher than mortality rates from infectious and parasitic diseases^1^.

In the country, the NCD in 2007 accounted for 72% of causes of death and affected more individuals that belong to vulnerable groups, such as the elderly^2^. Over the past decade, cardiovascular diseases (CVD) accounted for 50% of the mortality of all the NCD^3^. According to data from the Ministry of Health, NCD corresponded to the first cause of death in Brazil and accounted in 2008 for 40.8% of deaths of individuals aged 60 or older^4^.

Although CVD are the leading cause of death in Brazil, few studies have addressed the spatial and temporal distribution of mortality caused by them, especially regarding the elderly age group. Mortality from CVD is a phenomenon that has different risk factors, from behavioral and social factors to genetic ones and, therefore, one can infer that their distribution can be shown in different ways, as the context in which different population groups are inserted is variable. In this sense, it can be observed that the territory configurations, as well as the process of urbanization, have a direct impact on the way several population groups deal with this group of diseases^5^.

From this perspective, this study aimed to describe the geographical distribution of mortality from CVD in the elderly population in Brazil by gender, in the five-year period of 1996 to 2000 and from 2006 to 2010, and compare them in both periods. The search for a better understanding of the spatial and temporal distribution of these rates is critical for planning evidence-based sustainable public policies. This set of information can contribute to a better control and prevention of CVD, as it supports the achievement of more targeted actions for each country region, aiming thereby to reduce health inequalities.

## Methods

This was an ecological study, of which the area analysis units were the Brazilian states, which constitute 27 elements in the total sample. Data considered in the study are covered by the five-year periods of 1996-2000 and 2006-2010.

The study population was a group of elderly residents in Brazil who died from CVD in the analyzed period. For inclusion in the study, it was considered an elderly any individual aged ≥ 60 years^6^.

Data were obtained from the Data Processing Department of the Unified Health System (DATASUS), from the mortality information system (SIM). These data are grouped by SIM through the records of its legal instrument collection, the death certificate (DC). This information is available on the Internet for free consultation as data amassed by municipalities, that is, they were not individually and nominally collected. In this sense, there is no possibility of physical or moral damage from the individual's and community’s perspective, as the principles contained in Resolution 466 of 12 December 2012 were followed. Therefore, this article did not require approval by the Ethics Committee of Universidade Federal do Rio Grande do Norte (CEP-UFRN).

The outcome variables were the adjusted mortality rate from cardiovascular diseases (MRCD) in female elderly (MRCDf) and male (MRCDm) for each state. MRCD is calculated by the ratio of the number of elderly deaths from CVD by gender in Brazil in the assessed period and the elderly population in Brazil in the same period, by gender and per thousand inhabitants.

It is appropriate to clarify that the present investigation was based on the rate adjusted by the year 2003 population (corresponding to half of the study period - five-year periods of 1996-2000 and 2006-2010), as well as by age groups detailed by five-year intervals, from 60 years to 80 years and older, according to the standardization established by DATASUS.

We chose to perform, simultaneously, the comparison of mortality rates from III-defined causes (IDC) in both periods, to better demonstrate the information qualification process during the assessed period. Thus, a similar methodology was used to obtain adjusted rates of mortality from IDC in male and female elderly individuals.

Therefore, for comparative analysis, data were selected for two five-year periods, the first from 1996 to 2000, and the second from 2006 to 2010. The necessary population data to calculate the MRCD for each municipality were obtained from the Brazilian Institute of Geography and Statistics (Instituto Brasileiro de Geografia e Estatística - IBGE), available in DATASUS site. The TabWin software was used for data tabulation and calculation of mortality rates. The analysis of this coefficient in two different five-year periods allowed this study to assess changes in the epidemiology of mortality from CVD in Brazil, focusing on their geospatial characteristics.

This rate was calculated for each year of the two assessed five-year periods. Then, for each five-year period, using arithmetic mean, we obtained the mean mortality rate from cardiovascular diseases (MMRCD) per thousand individuals for each state of Brazil. This MMRCD was then distributed spatially to carry out exploratory and geostatistical analysis.

The IBGE cartographic shape was used in the study, which was obtained from its site. Initially, thematic maps were built for the two five-year periods, a phase that consisted in the exploratory analysis of spatial data. Their production was carried out using the SIG TerraView 4.2.0. program, in which the distribution amount was divided into five ranges for the legend, through the "equal step" division for the second five-year period, which was the basis for the first period distribution. At this point, the gray scale was chosen for visual comparison. At the time when the legend was created, which was carried out using a color gradient, the darker color represented the group of municipalities with the worse situations.

The spatial autocorrelation was calculated using the free Software TerraView 4.2.0 through the global Moran index for the MMRCD distribution in both analyzed periods. The value of the global Moran index ranges from -1 to 1. Values close to zero indicate lack of spatial autocorrelation; positive values indicate positive spatial autocorrelation; and negative values indicate negative autocorrelation. Subsequently, the standard analysis of the spatial distribution and the possible cluster formation was performed. For this, we used the local index of Moran-LISA, in order to map the intensity of clusters, considering a p value < 0.05 as statistically significant. The representative map of this situation is the Moran Map.

## Results

The overall population of elderly individuals in Brazil varied by 17.24% in the first analyzed five-year period, totaling 14,536,029 in 2000. Regarding the period between 2006 and 2010, there was a 30.57% increase in the elderly population, i.e., in the end of the last year of the second five-year period, there were 20,590,599 Brazilians aged 60 and older in absolute numbers. In relative terms, 7.86% of the population of the country was elderly in 1996 and, after 15 years, this number increased to 10.79%. Regarding the elderly population divided by gender, there was an increase between 1996 and 2010 of 38.22% for males and 41.03% for females.

In the period of 1996-2000, there were 4,629,638 deaths in Brazil, of which 53.8% occurred in individuals aged 60 and older. In the years 2006-2010, 5,396,557 records of deaths were released by SIM, with 60.5% being related to elderly individuals. Converting this analysis to the causes related to Chapter IX - Circulatory Disorders of the International Classification of Diseases and Health-Related Problems (ICD-10)^7^, it is possible to see that such diseases accounted for 27.51% of deaths in the general population and 37.42% among the elderly, in the first five-year period. For the second period, these numbers were respectively 29.19% and 37.17%.

**Graph 1:**
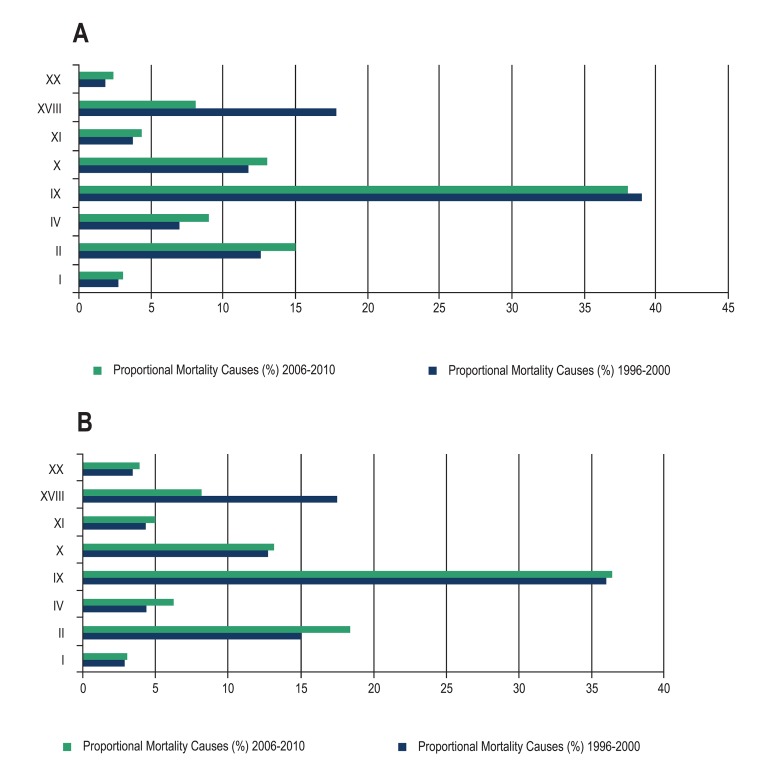
(A) Proportional mortality among female elderly in Brazil, divided by cause of death in five-year periods, from 1996 to 2000 and 2006 to 2010. (B) Proportional mortality among male elderly in Brazil, divided by cause of death in five-year periods, from 1996 to 2000 and 2006 2010. Chapter I: infectious and parasitic diseases; Chapter II: neoplasms; Chapter IV: endocrine, nutritional and metabolic diseases; Chapter IX: circulatory system diseases; Chapter X: respiratory diseases; Chapter XI: digestive diseases; Chapter XVIII: poorly-defined causes; Chapter XX: External causes. Source: Department of Informatics of the Unified Health System (DATASUS) / Ministry of Health.

Regarding the eight leading causes of death, Graph 1 shows the proportional mortality of the elderly, by gender, in both studied periods. In both genders, in the first five-year period, the three main causes of death corresponded to diseases of the circulatory system in the first place, IDC in the second, and neoplasms in the third; in the second five-year period, the top three were: diseases of the circulatory system, cancer and respiratory diseases.

Regarding the categories of most prevalent cause of CVD in Brazil, it can be observed that in the first five years, more than 50% were caused by the following conditions: acute myocardial infarction (21.18%), CVA (19.50 %), heart failure (13.43%) and chronic ischemic heart disease (6.09%). In the second five-year period, it was observed that the most prevalent were: acute myocardial infarction, with 22.05%; hemorrhagic or ischemic CVA, with 15.86%; heart failure, with 9.64% and primary hypertension, with 6.64%.

Table 1 shows the distribution of MMRCD in the elderly, per thousand inhabitants, by gender, in the Brazilian states in the period from 1996 to 2000 and from 2006 to 2010. The highest rates in the first five year period was concentrated in the South and Southeast states. The lowest value found in Brazil was observed in the state of Maranhão (4.24% for females and 5.32% for males), in the Northeast, and the highest in the state of Paraná (19.78% for females and 23.07% for males), in the South.

**Table 1:** Distribution by gender of the mean mortality rate from cardiovascular disease (MMRCD) in the elderly, per thousand inhabitants, in the Brazilian states in the periods 1996-2000 and 2006-2010

**Federation Unit**	**First 5-year period (A)**		**Second 5-year period (B)**		**Delta (B-A)**	
	**Female**	**Male**	**Female**	**Male**	**Female**	**Male**
Rondônia	13.56	14.15	11.75	13.12	-1.81	-1.03
Acre	8.31	9.32	9.58	11.96	1.27	2.64
Amazonas	8.02	8.94	7.90	9.86	-0.12	0.92
Roraima	11.03	15.87	9.49	12.72	-1.54	-3.15
Pará	8.69	9.46	8.74	10.72	0.04	1.26
Amapá	9.76	12.97	6.02	8.72	-3.73	-4.25
Tocantins	8.33	9.56	13.04	14.54	4.71	4.98
Maranhão	4.24	5.32	9.84	13.54	5.60	8.22
Piauí	6.59	8.43	14.68	17.83	8.09	9.39
Ceará	8.19	9.31	11.04	13.61	2.85	4.30
Rio Grande do Norte	7.44	8.69	9.99	12.67	2.55	3.98
Paraíba	5.43	6.00	11.99	14.00	6.56	8.00
Pernambuco	12.67	14.42	13.51	15.35	0.84	0.93
Alagoas	9.04	10.47	12.71	15.32	3.68	4.85
Sergipe	7.96	8.58	11.96	13.32	4.01	4.74
Bahia	8.10	9.01	8.73	10.03	0.63	1.02
Minas Gerais	13.76	16.25	9.84	12.03	-3.93	-4.21
Espírito Santo	13.63	17.46	12.50	15.89	-1.13	-1.58
Rio de Janeiro	17.14	22.27	11.36	15.77	-5.78	-6.50
São Paulo	18.00	22.00	11.51	14.96	-6.49	-7.04
Paraná	19.78	23.07	12.46	15.45	-7.32	-7.61
Santa Catarina	16.36	19.41	21.92	13.50	5.57	-5.91
Rio Grande do Sul	17.56	19.45	11.88	14.64	-5.69	-4.81
Mato Grosso do Sul	16.19	19.26	12.67	16.47	-3.52	-2.79
Mato Grosso	14.80	16.44	11.66	14.30	-3.14	-2.14
Goiás	14.41	15.54	11.31	12.99	-3.10	-2.55
Distrito Federal	17.25	21.35	9.63	14.29	-7.62	-7.05

Source: Mortality Information System (SIM). Department of Informatics of the Brazilian Unified Health System (DATASUS)/Ministry of Health, 2015.

In the period 2006-2010, the lowest rate was found in the state of Amapá (6.02% for females and 8.72% for men) and the highest in the state of Santa Catarina, for elderly women (21,92%) and Mato Grosso do Sul, for males (16.47%). A significant decrease was observed regarding results found in the South, Southeast and Midwest regions. In contrast, the North and Northeast regions showed significant increase in their rates, with emphasis on the states of Piauí, Paraíba, Maranhão and Tocantins, for both genders.

**Figure 1:**
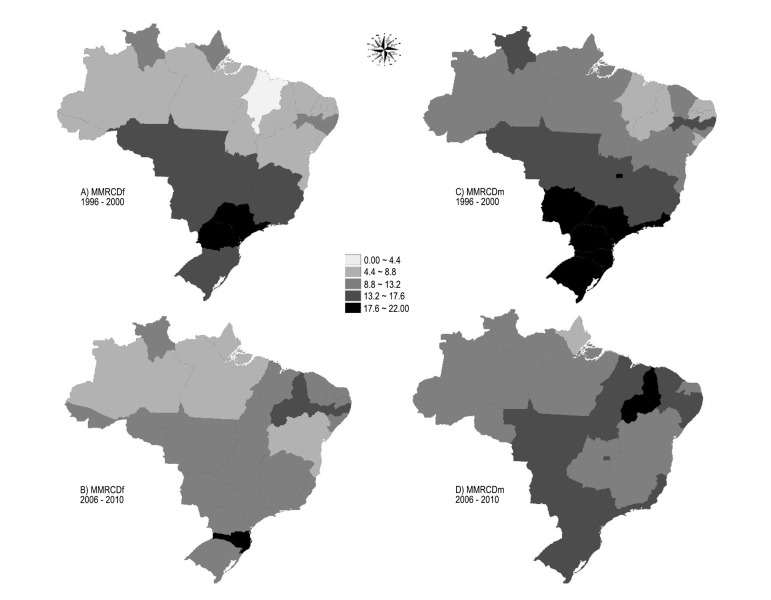
Thematic maps of the mean mortality rate from cardiovascular diseases (MMRCD) per thousand inhabitants in each Brazilian state and gender in the periods from 1996 to 2000 and from 2006 to 2010. Natal (RN), Brazil, 2015. Source: Department of Informatics the Unified Health System (DATASUS)/ Ministry of Health, 2015.

The analysis concerning the distribution of MMRCDf and MMRCDm in the Brazilian states is shown in Figure 1. The results were obtained from the mean rate of deaths from cardiovascular diseases in the elderly, in the periods 1996-2000 and 2006-2010, and the significance test for the global Moran's index, under the null hypothesis of absence of spatial autocorrelation.

It was observed that the global Moran’s index for the first five years was 0.225048 for females and 0.209145 for males, with p = 0.09 and 0.16, respectively. In the second five-year period, the value was 0.0887927, with p = 0.21 for elderly women and 0.0842536 and p = 0.21 for men older than 60 years.

**Figure 2:**
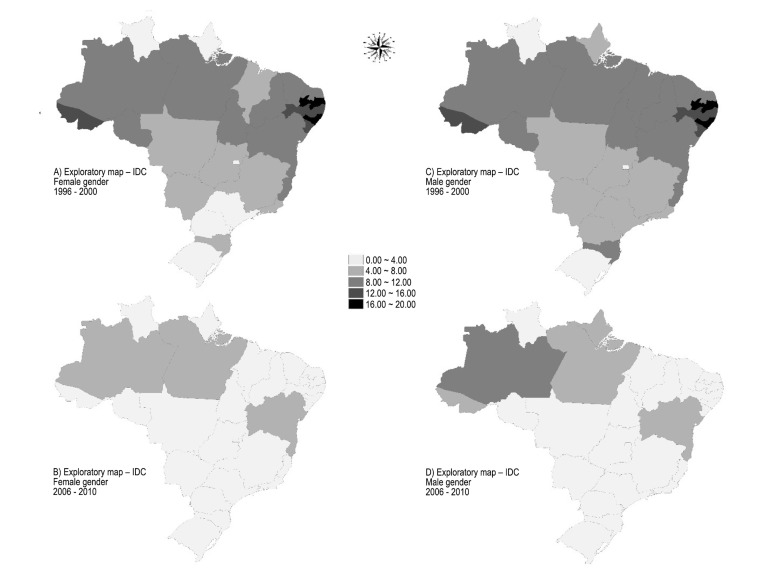
Thematic maps of the mean rate of mortality from ill-defined causes (IDC) per thousand inhabitants for each Brazilian state and by gender in the periods from 1996 to 2000 and from 2006 to 2010. Natal (RN), Brazil, 2015. Source: Department of Informatics of the Unified Health System (DATASUS) / Ministry of Health, 2015.

In order to support the results obtained by the aforementioned analysis, the characterization of deaths in the elderly was performed using an analogous methodology, by gender and Brazilian Federation unit, classified as ill-defined causes (IDC) (Chapter XVIII of ICD 10). The global Moran’s index values found for elderly women and men, in the first five-year period were, respectively: 0.388822 (p = 0.01) and 0.335994 (p = 0.04). For the second five-year period, the values were 0.06128 (p = 0.38) and -0.00415266 (p = 44). Distribution of deaths from IDC can be seen in Figure 2.

**Figure 3:**
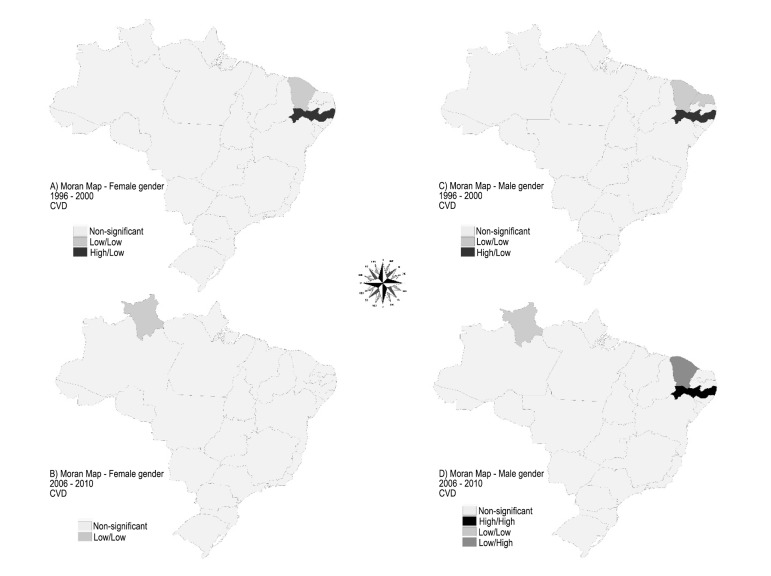
Moran Map (significant) of spatial autocorrelation of the variable mean mortality rate from cardiovascular disease by gender, per thousand individuals for each state in Brazil, in the periods 1996-2000, and 2006 to 2010. Natal (RN), Brazil, 2015. CVD: cardiovascular disease; Source: Department of Informatics of the Unified Health System (DATASUS) / Ministry of Health, 2015.

To create the maps depicted in Figure 3, we used the interpretation capabilities of the Moran Map, which allows the visualization of the statistically significant spatial autocorrelation areas^8^ and identify the location of homogeneous regions consisting of states with spatial association, regarding MMRCDf and MMRCDm. Thus, the Federation units were classified according to their location in relation to the Moran scatter plot quadrants: the quadrants 1 (high-high) and 2 (low-low) indicate areas with positive spatial association, i.e., the values were similar to those shown for neighboring states; quadrant 3 (high-low) and 4 (low-high) showed that the results did not follow the global trend and therefore had a negative spatial association, as there were neighbors that had discordant values^8^.

Therefore, in the first five-year period, in relation to the outcome variables, clusters were formed in the states of Ceará and Pernambuco for the female gender and in Rio Grande do Norte, Ceará and Pernambuco for the male gender. In the second five-year period, the autocorrelation was observed in the states of Roraima, for the group of elderly women, and Roraima, Ceará and Pernambuco, in relation to males.

**Figure 4:**
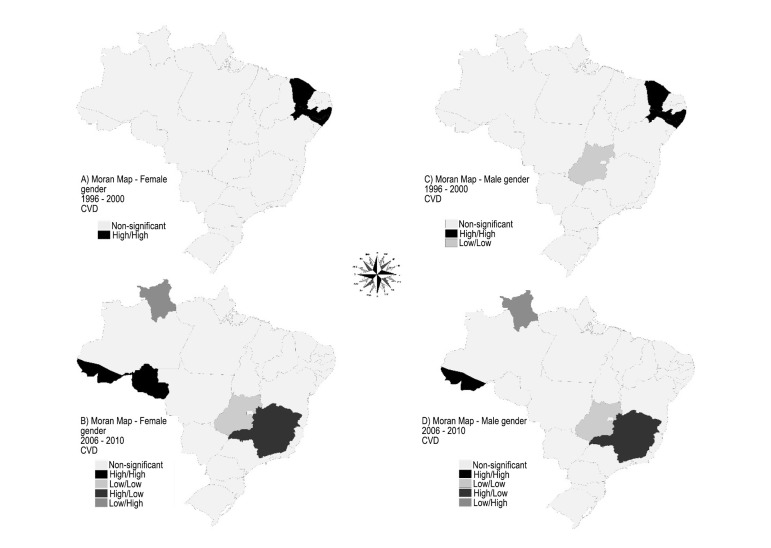
Moran Map (significant) of spatial autocorrelation of the mean mortality rate from ill-defined causes (IDC) by gender, per thousand individuals for each state in Brazil, in the periods 1996-2000, and 2006 to 2010. Natal (RN), Brazil, 2015. Source: Department of Informatics of the Unified Health System (DATASUS) / Ministry of Health, 2015.

It is noteworthy the fact that for the spatial autocorrelation analysis performed for deaths classified as IDC, as seen in Figure 4, the methods described for the MMRCDf and MMRCDm variables were used. Therefore, clusters were formed, in the first five-year period, in Ceará and Pernambuco for both genders. In the second five-year period, the autocorrelation was observed in the states of Roraima, Acre, Rondonia, Goias and Minas Gerais for the elderly females, and Roraima, Acre, Goias and Minas Gerais for males.

## Discussion

Based on the results, it was observed that in the first five-year period, the highest MRCD were mainly concentrated in the South and Southeast regions. In the second five-year period, there was a considerable increase in the rates observed in the Northeast, as well as a decrease in the rates in the South and Southeast regions. There was also a slight increase in mortality rates in the North region.

The emergence of a cluster in the states of Rio Grande do Norte, Ceará and Pernambuco, as seen in the Moran Map for both genders, in the period 1996-2000, allowed the identification of a positive spatial association of MMRCD, indicating the similarity of this rate between those states and their neighbors. Thus, a homogeneous area in the Northeast was observed, characterized by high levels of MMRCD that appeared in the second five-year period.

The literature shows that mortality statistics, as shown in this study, are the most often used to obtain health status information of a population and to plan necessary actions for health promotion. However, it is of utmost importance to also discuss the proportion of deaths attributed to IDC, as it constitutes one of the indicators used to assess the quality of that information and the correct trend of the mortality analysis^9^.

At the stratification of deaths in the elderly population in Brazil for the period of 1996-2000, the causes identified as ill-defined encompassed the second overall position, with a total of 17.64% of notifications. For the second five-year period, there was substantial improvement, relocating mortality from undetermined causes to the fourth position (8.10%). Therefore, the lowest proportion of notifications from Chapter XVIII of ICD-10 (CMD) indicates more accurate statistics on mortality^10^.

In the country, the highest number deaths from ill-defined causes concentrated in the age group older than 60 years; that is, regarding the data for 2005, 67.2% of deaths from IDC corresponded to this population group. One explanation for the high proportion of deaths from IDC is the difficulty in establishing the underlying cause of death in the elderly. This is probably due to the presence of multiple diseases in the elderly and the influence of age on the clinical expression of signs and symptoms for the correct diagnosis of the underlying cause of death^11^. In this sense, the data obtained in the first five years for the analysis carried out on deaths from IDC in the elderly corroborate the above statements, as they show statistically significant results (p < 0.05).

One question to be assessed is the increased prevalence of CVD in the North and Northeast states during the study period. This fact was possibly related to the information production qualification, both regarding the collection and sending of data to the health management central level. Studies have shown that over the past three decades, advances in health information systems used in Brazil were supported by the development of computer technology and the training of Health Secretariat employees^12,13^.

The mortality information is compiled from SIM of the Ministry of Health, which was designed in 1975 and initially covered only some Brazilian states, which already held the collection of this information^12^. Another milestone related to the development of the mentioned information system was the creation of the current design of the DO, along the development of a new computerized application, which was first used in 1999^14^.

The information on mortality is obtained from the SIM of the Ministry of Health, designed in 1975 and that initially covered only a few Brazilian states, which already performed this information collection^12^. Another milestone related to the development of the aforementioned information system was the creation of the current design of the DC, along with the development of a new computer application, which was first used in 1999^14^.

In 2004, the Health Surveillance Secretariat of the Ministry of Health included the program Percentage Reduction of Death from Ill-defined Causes in the Multi-Year Plan 2004-2008 and the "Investigation of the cause of death” form was standardized. This program included the data provided by medical or health professionals, or those obtained from medical records and the results of additional tests, to also ensure a more accurate recording of information on the causes of death^15^.

In addition to this investigation, in March 2008, the Ministry of Health launched a project to implement the verbal autopsy in the country as a method to investigate deaths from IDC, so that its analysis would allow the physician to identify the sequence of events that led to the death. Several international studies using verbal autopsy methodology also observed changes in the structure of causes of death, decrease in IDC and identification of external causes, with the most frequent diseases being allocated in the chapter of circulatory diseases and external causes - results that are similar to those observed in this study^16^.

This phenomenon is called the "paradox of information", which is characterized by the relocation of deaths from IDC to other chapters of ICD-10. That is, the variation in the proportion of death notifications from IDC can modify the temporal series of mortality rates for certain groups of causes^17^. In this regard, the absolute increase in the number of deaths from diseases of the circulatory system is closely associated to the decrease in notifications originating from Chapter XVIII.

Another factor to be considered is the significant increase in mortality from CVD among the elderly in the North and Northeast states of the country, a trend less intense in the South, Southeast and Midwest regions, where the increases were discreet. Such regional variation is influenced by the fact that primary and secondary prevention would be more appropriate in more developed regions, with better control of risk factors for CVD, such as smoking, dyslipidemia, diabetes and systemic hypertension^9^.

In a study carried out between 2000 and 2009 in Brazil, it was clear that the coverage provided by the family health strategy was associated with a reduction in MRCD (acute myocardial infarction and CVA). However, it is noteworthy that this study evaluated 1,662 of the 5,507 Brazilian municipalities and used as an exclusion criterion the municipalities with high mortality rates from IDC. Therefore, their results are valid for municipalities where the quality of information is better^18,19^.

Another relevant aspect is related to the distribution of medical professionals registered in the Regional Councils of Medicine in their physician per thousand inhabitant ratio. In this regard, the North and Northeast regions have the lowest proportions, 0.98% and 1.19%, respectively, below the national average, which is 1.95%^20^. That is, the North and Northeast regions have higher limitation regarding health services provided to the population, a fact that also corroborates the increase in MRCD in the study.

Regarding the aspects related to the epidemiological transition, it is possible to estimate that, in general, it occurs together with socioeconomic transformations and, therefore, it shows major demographic differences^21^. Hence, the majority of published studies provide evidence of the association between social inequalities and morbimortality. Brazil is the tenth most unequal country in the world in terms of income distribution; even though changes in the economy have resulted in improvements in this regard, they do not seem to have been able to reduce mortality inequalities. Hence, important differences persist in the distribution of morbidity and mortality, both between different Brazilian states and within the same state^22^.

It is worth mentioning that this study has as limiting factor the use of data collected by a Federation state, which can conceal the heterogeneous distribution of deaths and, therefore, mask relevant intrastate differences. Furthermore, the use of secondary data record is subject to several data recording errors and underreporting.

Another limitation is due to the "ecological fallacy" in which, due to the effects of data aggregation and scale, the results found for a population cannot be repeated at the individual level. In this study, the mortality analysis was based only on the cause of death and there was no analysis of multiple causes; thus, there may be an underestimation of cardiovascular mortality, especially among the elderly, which may have several comorbidities.

## Conclusion

Based on the analyses, it was observed that the proportion of mortality from circulatory system diseases in the elderly, in the 2006-2010 period, decreased significantly in the South, Southeast and Midwest states, and showed a considerable increase in the North and Northeast regions. These results are consistent with geographical clusters obtained in the aforementioned period, during which spatial autocorrelation was observed between the states of Rio Grande do Norte, Ceará, Pernambuco and Roraima.

These findings can be explained by the information qualification, reallocation of deaths from ill-defined causes and improved health care. Added to these, other issues were also evaluated, such as changes in the socioeconomic situation of the country, mainly regarding the phase of epidemiological transition in which Brazil currently is, with decreased morbidity and mortality from infectious and parasitic diseases, and increase in the number of deaths due to chronic non-communicable diseases and external injuries.

Effective planning of health promotion actions originates from the knowledge of a population’s health status, based on mortality statistics. The quality of information on the causes of death is, therefore, essential. The search for a better understanding of the spatial and temporal distribution of these rates is critical for the planning of evidence-based sustainable public policies. Therefore, the study may contribute to better control and prevention of cardiovascular disease, as it supports the achievement of more targeted actions for the different regions of the country, thus aiming to reduce health inequalities.

The study was carried out with the authors’ own resources and also received a scientific initiation research grant (PIBIC) from Pró-Reitoria de Pesquisa da Universidade Federal do Rio Grande do Norte (PROPESQ-UFRN).
